# Coexistence and Social Exclusion of Venezuelan Migrants in Lima, Perú: A Psychosocial Approach

**DOI:** 10.11621/pir.2024.0405

**Published:** 2024-12-01

**Authors:** Rosa M. Cueto, Liz S. Ayma, César A. Llanco, Fiorella Acedo, Camila Loli

**Affiliations:** a Pontifical Catholic University of Peru, Lima, Perú

**Keywords:** migration, exclusion, intergroup relations, Peru

## Abstract

**Background:**

An understanding of the dynamics of intergroup relations, particularly in relation to migrant populations, is of critical importance in a variety of societal contexts. This study examines the relationship between conservatism (Social Dominance Orientation, SDO), intergroup dynamics (stereotypes and intergroup emotions), and social distance (openness to coexistence and tendencies toward exclusion) within the Venezuelan migrant population residing in Lima Metropolitana.

**Objective:**

The objective of this study was to investigate the relationships between conservatism, intergroup dynamics, and social distance among Venezuelan migrants in Lima Metropolitana. A sample of 395 residents was utilized for this study.

**Design:**

The study employed a correlational research design to examine the relationships between the variables of interest. Correlation analyses were conducted to assess the associations between variables. Path analysis was utilized to explore the underlying mechanisms. Furthermore, group comparisons were conducted to examine differences in attitudes and perceptions across various demographic groups.

**Results:**

The findings indicated that there were significant associations across conservatism, intergroup emotions (both negative and positive), stereotypes, and the tendencies toward exclusion and coexistence. Notably, younger participants exhibited more inclusive ideological and intergroup indicators. Moreover, the path analysis demonstrated the pivotal influence of the morality stereotype and ideological dimensions on the integration and exclusion of migrants.

**Conclusion:**

The study highlights the significance of cultivating favorable stereotypical perceptions of migrants and emphasizes the impact of age and ideological factors in creating conducive conditions for migrant integration. A contextual understanding of these results underscores the necessity for inclusive policies and interventions that foster social cohesion and integration within communities that have received migrants.

## Introduction

Migration involves the movement of individuals from one territory to another to settle temporarily or permanently (International Organization for Migration [IOM], 2019a). Globally, an estimated 281 million people have migrated to other countries, representing a growth rate of 2.5% between the years 2015 to 2020 (Migration Data Portal, 2021).

A sense of precariousness in the place of origin is linked to this process, driven by the expectation of creating a personal or group project aiming at change and social ascent (Gutiérrez et al., 2020). According to the IOM (2020), migration is a politically significant phenomenon that should be examined from both a rights-based approach and a perspective that considers the sociocultural and civic-political contributions of migrant populations as well as their impact on the development of the receiving host country. The characteristics of migration processes reflects the legal and political frameworks of both the place of origin and destination.

Due to their institutional and legal powerlessness, migrants, especially those in irregular situations, face several risks and difficulties including human trafficking and sexual/or labor exploitation that may occur along their migration path and regions of destination. These unprotected conditions are often followed by experiences of xenophobia and discrimination, which end up hindering their social integration (IOM, 2020). Evidence also indicates that those who migrate because of violence and/or poverty are even more vulnerable to exclusion, as their initial situation may be exacerbated by hostility experienced in the host country (IOM, 2019b).

A highly relevant migration process in Latin America today is that of the Venezuelan migrant population, driven by the severe political and economic instability in that country. The Interagency Coordination Platform for Refugees and Migrants from Venezuela (R4V, 2024) reports that 1.54 million of the 7.8 million Venezuelan migrants reside in Peru. With a relatively equal distribution by gender and a majority holding higher education qualifications, the Venezuelan refugee and migrant population in Peru increased by 41.5% between 2018 and 2022 (Instituto Nacional de Estadística e Informática [INEI], 2023).

The public’s image of immigration in Peru has been greatly impacted by the rise in the number of Venezuelan migrants, resulting in a conflict between the economic advantages of immigration and social and cultural issues that arise (Napan, 2023). Eight out of ten Peruvians oppose having more Venezuelans in their personal social circle, with rejection sentiment stronger in rural areas, among women, older adults, and among poorer socioeconomic backgrounds (Amaya & Elguera, 2023). Furthermore, with an average score of 4.15 out of 10, sympathy for Venezuelan refugees is low, indicating a distant and reserved attitude toward this group.

Venezuelan migrants in Peru are a group that experiences social vulnerability (Berganza & Solórzano, 2019; IOM, 2022). These situations include economic insecurities, housing overcrowding or difficulty finding housing, health or education precariousness, and difficulty accessing work, demonstrating the Peruvian State’s shortcomings in supporting their integration (Berganza & Solórzano, 2019). In addition, immigrants are exposed to exploitation, discrimination and xenophobia (Freier et al., 2021), highlighting the importance of a psychosocial exploration into the dynamics and intergroup relations that affect the integration of the Venezuelan population in this country.

Intergroup relations develop from the process of social categorization, a mechanism that organizes and classifies social stimuli into social categories facilitating an individual’s differentiation from members of other groups (outgroup) and their identification with members of their own group (ingroup) (Abrams & Hogg, 1998; Fiske, 1998; Stangor, 2009; Tajfel, 1982; 1984). The need of individuals and groups to maintain a positive self-image derives from endogroup characteristics engendering social comparisons between the endogroup and the exogroup. This leads to the accentuation of certain intergroup differences and their valuation in favor of the ingroup, especially socially valued characteristics (Abrams & Hogg, 1998).

The characteristics commonly attributed to social categories and their members are called stereotypes (Castro, 2006). Stereotypical attributes help distinguish between endogroup and exogroup members, acknowledging differences between exogroups, justifying intra- and intergroup actions, validating intergroup differences and dominance, and understanding intergroup tensions and conflicts (Fiske et al., 2003; Jost et al., 2004; Oldmeadow & Fiske, 2007). However, stereotypes will have a detrimental effect if they adopt negative connotations, which is often the case for those exogroups considered to be of lower status and/or perceived as a threat to the ingroup and/or to the status quo (Bodenhausen et al., 2000; Castro, 2006; Stangor, 2009). In these cases, negative stereotypes may result in negative expectations, misjudgments or discriminatory behaviors toward members of negatively stereotyped groups (Fiske, 2012).

Moreover, studies on intergroup relations suggest emotions may be even more effective than stereotypes in predicting behavioral tendencies toward a given group (Cuddy et al., 2007). Intergroup emotions are generated following an evaluation of the intergroup context, based on the perception of potential harm or benefit posed by the exogroup or its members (Mackie et al., 2000). Intergroup emotions are often empirically associated with two stereotypical dimensions attributed to exogroups: warmth and competence. The first dimension refers to the stereotypical attributes associated with the social behaviors and relationships of individuals in each group, allowing inferences about their disposition toward cooperation or potential intergroup conflict (Fiske et al., 2003). The second dimension is related to characteristics linked to the general efficacy and capacity of group members to achieve the goals they set for themselves (Cuddy et al., 2007; Gómez, 2019; Rodríguez et al., 2013).

Both stereotypical dimensions (warmth and competence) form a double-entry model, wherein groups perceived to be competent and warm inspire emotions of pride and admiration; those that are not would inspire contempt; groups perceived as warm but not competent would inspire sympathy; and those that are seen as competent but not warm inspire envy (Cuddy et al., 2007; Fiske et al., 2002).

Studies in the Latin American context find an additional stereotypical dimension: morality, characterized by traits such as honesty and tolerance (Espinosa et al., 2016). High levels of moral characteristics are usually attributed to less-favored groups, often those of lower status, mainly in contexts of discrimination and exclusion (Espinosa et al., 2016; Guillén et al., 2018), as is often the case with migrant groups (Contreras & Saldívar, 2018). Likewise, evidence shows that groups considered lower status tend to generate emotions such as hostility, anger, contempt or sadness (Espinosa et al., 2007).

Conversely, conservative political ideology, which lies at the core of intergroup relations, is characterized as a set of values, attitudes, and beliefs that allow an individual to understand the social and political world, assess it, and act to preserve the status quo; this perspective facilitates the analysis of intergroup behavior (Jost et al, 2003; Jost et al., 2004). Accordingly, the widespread inclination to feel compelled to support social hierarchies that maintain intergroup inequalities is explained through social dominance orientation (SDO), a component of conservative political ideology (Pratto et al., 1994). Beliefs regarding each person’s proper place in society are based on their social category of membership, which is typically tied to their ethnicity of origin, and economic standing, as well as the conventional traits that are associated with these categories (Duckitt & Sibley, 2007). Thus, the mere existence of social categories perceived as low status will be perceived as a threat to the social order, which encourages the most conservative ideological sectors to prejudge and discriminate against members of these groups (Rottenbacher et al., 2011).

According to numerous studies, the SDO promotes prejudice against members of socially disadvantaged groups (Duriez & Van Hiel, 2002; Sidanius & Pratto, 2004). SDO has been found to be a strong predictor of prejudice against migrants, particularly when they are perceived as competent in the workplace (Cohrs & Asbrock, 2009; Vezzali & Giovanni, 2010). This is because a receiving society’s perception of migrants as a threat to labor market access, impedes their social and cultural integration (Blouin, 2019; IOM, 2022).

In accordance with research on intergroup interactions in the context of Venezuelan migration to Peru, some segments of the Peruvian population express mistrust and anxiety toward them, which has occasionally resulted in violent crimes and discriminatory practices (Loayza, 2020). This also aligns with the unfavorable intergroup portrayals and assessments of this social category that other writers have documented (Berganza & Solórzano, 2019; Diaz et al., 2018). In this regard, some studies find that the social category of Venezuelan migrants is stereotyped as being not very competent and lazy. Additionally, they are blamed for generating unfair competition for native Peruvians, as they are associated with labor precariousness and informality (Blouin, 2019). According to Koechlin et al. (2021), the presence of Venezuelan communities in Lima, the city with the highest concentration of Venezuelan migrants, is linked to higher levels of crime, disorder, and insecurity.

However, there is also evidence of a more positive perception of this social category, linked to stereotypical characteristics of competence, warmth and morality, which are linked to less negatively charged affective and emotional indicators (Cueto, 2017; Robinson & Espinosa, 2021). Similarly, a study conducted in Arequipa revealed that the levels of prejudice towards the Venezuelan community were significantly lower among younger age groups, who also showed greater openness to their integration into Peruvian society (Álvarez & Chávez, 2018).

Based on the above, conservative ideology establishes the framework for general beliefs about the dynamics of intergroup relations (Jost et al., 2003; Jost et al., 2004). This includes how people think, value and perceive emotions in relation to particular social categories and their members, as well as how they assign them a certain level of status based on the stereotypical traits and social valuations attributed to them (Duckitt & Sibley, 2007; Rottenbacher et al., 2011). Hence, in the case of the migrant population, it is relevant to study the extent to which political ideology serves as an antecedent to intergroup variables (stereotypes and intergroup emotions) that hinder integration and social coexistence, considering that this social category is usually perceived as low status (Berganza & Solórzano, 2019; Blouin, 2019; Contreras-Ibañez & Saldívar, 2018; Diaz et al., 2018).

In the case of the Venezuelan migrant population in Peru, most recent studies focus on general immigration issues, but fail to offer an articulated analysis of the influence of conservative ideologies and particular intergroup dynamics on attitudes toward this community. By investigating these factors, the study seeks to improve understanding of the obstacles to the integration of the Venezuelan population in Peru, thereby contributing to the formulation of interventions that mitigate prejudice and promote social inclusion.

The central objective of this research is to analyze the influence of social dominance orientation (SDO) and intergroup dynamics (intergroup emotions and stereotypes towards exogroups) on attitudes of social distancing, specifically focusing on openness to coexistence and tendency toward exclusion of the Venezuelan migrant population, among residents of Metropolitan Lima. The first objective is to examine the relationships between ideological variables, such as the SDO, and attitudinal variables. This analysis aims to elucidate the way these ideological constructs shape attitudes towards Venezuelan migrants. Secondly, the study seeks to identify the specific emotions and stereotypes associated with the tendencies toward either exclusion or inclusion of this migrant population. This will be done by considering how emotional responses and stereotypical representations can influence the disposition of individuals towards the interaction or rejection of these groups. Finally, the third specific objective is to assess differences in attitudes toward Venezuelan migrants between age groups — those over and under 30 years old. This focus stems from the Peruvian context, where social conditions have led to varied responses to social phenomena based on age, distinguishing the so-called “bicentennial generation” from previous generations. This analysis aims to elucidate whether generational differences influence attitudes towards the integration or exclusion of Venezuelan immigrants and to ascertain the extent to which these differences may be related to underlying ideological and psychosocial factors.

The hypotheses of the present study aim to clarify the relationships between SDO, emotions and stereotypes about Venezuelan immigrants, as well as attitudes towards the inclusion or exclusion of this community in the Peruvian context. First, it is hypothesized that SDO levels will be positively correlated with negative emotions towards Venezuelan immigrants and inversely correlated with positive emotions toward them (H1). Additionally, an inverse relationship is expected between SDO levels and positive stereotypical attributes of Venezuelans and directly associated with tendencies toward exclusion and negative emotions toward this community (H2).

Conversely, it is proposed that openness to coexistence with Venezuelan immigrants will be inversely related to SDO and negative emotions and positively related to positive emotions toward them (H3). Similarly, it is expected that positive intergroup stereotypes and positive emotions towards Venezuelans will be directly related to greater openness to coexistence and inversely related to a tendency towards exclusion (H4). Finally, it is expected that participants under the age of 30 will have lower levels of prejudice and greater openness to coexistence with the Venezuelan community than those aged 30 and over (H5).

## Methods

### Participants

A total of 395 residents of Lima Metropolitana participated in the study, ranging in age from 18 to 75 years (M = 29.54, SD = 11.19). Of the total, 62.5% were women, 64.3% had higher education, 31.4% had secondary education, and 4.3% reported having another level of education. Additionally, 23% reported having children. Of those surveyed, 71.4% were born in Metropolitan Lima, 56.4% reported that their parents were born in Lima, and 83.8% had relatives living abroad. Finally, regarding political orientation, 17% identified as left-wing, 48.6% positioned themselves as center, and 34.4% expressed being right-wing.

The inclusion criteria required participants to be Peruvian, reside in Lima, and at least 18 years old. To facilitate accessibility to study participants, a non-probabilistic convenience sampling approach was implemented. To broaden demographic representation within the sample, selection criteria were used to ensure the inclusion of a variety of age groups. As part of the ethical criteria of the research, participants were informed of the study’s objective and asked for their permission to participate through informed consent, indicating that participation was strictly voluntary and confidential. They were also informed that the collected information would only be used for the purposes of the study and any dissemination in academic spaces would maintain anonymity.

### Measurement

#### Social Distance [SD] (Bogardus, 1947)

This scale has been adapted to measure the desired level of contact with Venezuelan immigrants. It consists of 7 statements, of which 5 correspond to the dimension of *openness to coexistence* (ie, “I would live with a Venezuelan person as a partner”) and 2 items refer to *tendency to exclude* (ie. “I would exclude Venezuelan people from my neighborhood”). The survey consists of a 7-point Likert response scale ranging from 1 (“completely disagree”) to 7 (“completely agree”). The first factor, *openness to coexistence*, showed a Cronbach’s α = .94, while the second factor, ‘tendency to exclude’, obtained a α = .8.

*Social Group Stereotype Scale (Espinosa et al., 2016)*

This instrument measures the stereotypical characteristics associated with different ethnic or social groups. For this study, the instrument included 20 items, each consisting of pairs of 20 positive adjectives and their respective antonyms, potentially representative of the Venezuelan social category. The response options are presented on a 7-point Likert scale, indicating the perceived proximity or distance of the social category to these attributes. The items are grouped into 3 dimensions: Competence (6 items, “Unsuccessful – Successful”); Warmth (7 items, “Sad/Melancholic – Happy”); and Morality (7 items, “Dishonest – Honest”). Cronbach’s α were found to be α = .90 for Skill, α = .86 for Warmth, and α = .94 for Morality.

### Differential Emotions Scale (Izard, 1991)

The Spanish version of Espinosa et al. (2007) was used to measure the intensity of 10 emotions towards a specific stimulus: in this case, the Venezuelan social category. A Likert scale was used, ranging from 1 (“not at all”) to 7 (“a great deal”). Cronbach’s α = .76 was reported for positive emotions, and α = .88 for negative emotions.

### Social Domination Orientation Scale (Sidanius & Pratto, 2004)

The Spanish version of the Sidanius and Pratto (2004) SDO scale was used (Cárdenas et al., 2010), consisting of 16 items with a Likert response scale ranging from 1 = “strongly disagree” to 7 = “strongly agree”. *Opposition to equality* and *group dominance* were the two dimensions utilized in the adaptation of the scale. The present study reported the following Cronbach’s α = .94 for opposition to equality and α = .84 for group dominance. Additionally, a satisfactory reliability coefficient was obtained for the overall scale: α = .89.

### Procedure and data analysis

An online survey form was designed using the Google Forms platform. The first section of the form presented the informed consent document, which indicated the voluntary, confidential, and anonymous nature of participation. A sociodemographic information sheet was included, as well as the previously described instruments. The form was disseminated through virtual means to Peruvian individuals over 18 years of age residing in Metropolitan Lima. After completing the questionnaires with the entire sample, data analysis was performed using the Statistical Package for Social Sciences (IBM SPSS) version 25.

The research had a non-experimental correlational design. First, normality analyses were conducted using the Kolmogorov-Smirnov test (N > 50). Additionally, histograms and box plots were reviewed. Although the Kolmogorov-Smirnov coefficient indicated a non-normal distribution, considering the skewness and kurtosis criteria (|Sk| < 2; |Kr| < 7), there were no signs of serious breach of normality (Curran et al., 1996).

Descriptive statistics were obtained for the sociodemographic data and applied scales. The Student’s t-test was used to compare the participants’ age groups. Pearson’s correlation coefficients were calculated. The effect size of the results obtained in the analyses was determined. The nature and magnitude of the relationships between the studied variables were analyzed in an integrated manner through path analysis using the AMOS v.26 program. Both dimensions of the social distance scale were considered as output variables.

## Results

*Table 1* shows the sample statistics for mean, standard deviation, minimum, and maximum. The mean values indicate moderately high scores for the dimensions of exogroup stereotypes and openness to coexistence. Positive scores are reported overall regarding attitudes towards the Venezuelan population and social interaction with them. On the other hand, low scores are identified for the dimensions of negative emotions towards Venezuelans and the dimension of tendency to exclude on the social distance scale. It is noteworthy that the score for sadness is quite close to the average score between the minimum and maximum scores on the scale.

**Table 1:** Descriptive data of the study variables

	M	SD	Sk.	Kr.
*SDO*				
1. Opposition to equality	2.59	1.50	.91	–.07
2. Group dominance	2.43	1.16	.86	.20
*National Stereotypes*				
3. Competent Venezuelans	4.84	1.02	–.55	.90
4. Warm Venezuelans	5.31	.90	–.51	.28
5. Moral Venezuelans	4.35	1.07	–.25	.25
*Intergroup Emotions*				
6. Joy	3.33	1.83	.16	–1.11
7. Surprise	3.24	1.75	.21	–1.03
8. Sadness–Grief	3.88	1.84	–.03	–1.09
9. Interest	3.26	1.64	.16	–.92
1. Anger	2.06	1.48	1.51	1.77
11. Disgust	1.83	1.38	1.80	2.63
12. Contempt	1.63	1.18	2.25	5.24
13. Guilt	1.68	1.16	1.79	2.75
14. Shame	1.60	1.20	2.35	5.55
15. Envy	1.36	.88	2.80	7.74
*Social Distance*				
16. Coexistence	5.32	1.78	–.97	–.29
17. Exclusion	1.76	1.30	2.18	4.68

*Note. Source: Own elaboration based on collected data. Min = 1, Max = 7*

### Contrast of means by age groups

The statistical analyses revealed significant differences between the age groups (15–29 years and 30 years and over) according to emotions and openness to cohabitation. In the category of emotions, the younger age group (15–29 years) showed significantly higher scores for the emotions of joy and guilt compared to the group aged 30 and over. In the category of openness to coexistence, significantly higher scores were also observed in the younger age group, suggesting a greater willingness to interact with the Venezuelan immigrant population (see *Table 2*).

**Table 2:** Comparison of means between groups by age*

	Bicentennial group (n = 265)		Non Bicentennial group (n = 130)		*t*(393)	p	*d*
	M	SD	M	SD			
*SDO*							
1. Opposition to equality	2.51	1.43	2.75	1.64	–1.40	.16	–.16
2. Group dominance	2.38	1.12	2.53	1.25	–1.25	.21	–.13
*Intergroup Stereotypes*							
3. Competent Venezuelans	4.88	.95	4.77	1.14	1.03	.30	.11
4. Warm Venezuelans	5.30	.93	5.34	.84	–.49	.63	–.05
5. Moral Venezuelans	4.38	1.00	4.29	1.22	.73	.47	.08
*Intergroup Emotions*							
6. Joy	3.52	1.79	2.96	1.85	2.86	.001	.31
7. Surprise	3.29	1.69	3.13	1.85	.83	.40	.09
8. Sadness–Grief	3.89	1.80	3.84	1.95	.28	.78	.03
9. Interest	3.35	1.61	3.08	1.70	1.54	.12	.17
1. Anger	2.08	1.42	2.02	1.59	.38	.70	.04
11. Disgust	1.84	1.33	1.82	1.47	.15	.88	.02
12. Contempt	1.64	1.14	1.62	1.27	.12	.91	.01
13. Guilt	1.75	1.21	1.52	1.02	1.99	.04	.20
14. Shame	1.59	1.22	1.61	1.18	–.12	.91	–.01
15. Envy	1.39	.92	1.29	.78	1.07	.29	.11
*Social Distance*							
16. Coexistence	5.54	1.60	4.87	2.02	3.31	.001	.38
17. Exclusion	1.70	1.21	1.88	1.47	–1.20	.23	–.14

*Note: According to our own elaboration based on collected data, individuals up to the age of 29 are classified as part of the bicentennial group.*

### Associations among the study variables

*Table 3* displays the associations found among the study variables. Concerning the SDO dimensions, a medium and inverse correlation was obtained between the dimension of openness to coexistence and opposition to equality, and an inverse and small correlation with group dominance. Regarding exogroup stereotypes, a strong positive correlation was obtained with the dimensions of competence and morality, while a small positive correlation was observed with warmth. Regarding intergroup emotions, a moderate positive correlation was found with joy and interest, and a small positive correlation with sadness and surprise. Additionally, a small negative correlation was reported with anger, disgust, and contempt.

**Table 3:** Study Variable Pearson Correlations

	1	2	3	4	5	16	17	18
*Social Dominance Orientation*								
1. Opposition to equality	–							
2. Group dominance	.24**	–						
*Intergroup Stereotypes*								
3. Competent Venezuelans	–.32**	–.26**	–					
4. Warm Venezuelans	–.23**	–.17**	.74**	–				
5. Moral Venezuelans	–.28**	–.30**	.82**	.65**	–			
*Intergroup Emotions*								
6. Joy	–.17**	–.06	.37**	.37**	.44**	.35**	–.15**	–.17**
7. Surprise	–.06	–.04	.24**	.24**	.29**	.17**	–.06	–.06
8. Sadness–Grief	–.22**	–.11*	.26**	.26**	.25**	.23**	–.06	.05
9. Interest	–.21**	–.16**	.39**	.36**	.40**	.31**	–.13*	–.07
1. Anger	.09	.14**	–.20**	–.09	–.23**	–.19**	.33**	–.06
11. Disgust	.20**	.22**	–.30**	–.14**	–.31**	–.25**	.36**	–.04
12. Contempt	.22**	.15**	–.30**	–.15**	–.30**	–.26**	.37**	–.05
13. Guilt	.14**	.02	–.06	–.05	.01	–.02	.05	–.11*
14. Shame	.17**	.11*	–.15**	–.05	–.14**	–.10	.14**	–.02
15. Envy	.21**	.04	–.08	–.04	–.03	–.04	.05	–.05
*Social Distance*								
16. Coexistence	–.43**	–.19**	.51**	.35**	.60**	–	.17**	–.23**
17. Exclusion	.24**	.36**	–.42**	–.25**	–.49**	–.46**	–	.07

Note: 18 = Age. Source, Own elaboration based on collected data, *p<.05; **p<.01

Lastly, a small negative correlation was found with age. The tendency to exclude dimension showed a small positive correlation with opposition to equality and a moderate positive correlation with group dominance. Regarding exogroup stereotypes, medium to low inverse correlations were found with the dimensions of competence and morality, and a small inverse correlation with warmth. As for intergroup emotions, a small inverse correlation was found with joy and interest, and a small direct correlation with shame, as well as a medium direct correlation with anger, disgust, and contempt. Finally, a moderate inverse correlation with openness to coexistence is reported.

In turn, opposition to equality showed a small direct correlation with group dominance, age, and emotions of disgust, contempt, guilt, shame, and envy. Additionally, it obtained moderate inverse correlations with the dimensions of warmth, morality, joy, interest, and sadness, and a moderate inverse correlation with the dimension of competence. The group dominance report showed small inverse correlations with the competence, warmth, interest, and sadness dimensions, and a medium inverse correlation with morality. Additionally, it found small direct correlations with the emotions of Anger, disgust, contempt, and shame.

The dimensions of the social group stereotypes scale (warmth, morality, and competence) showed a moderate positive correlation with the emotion of joy, and a small positive correlation with the emotions of surprise, sadness, and interest. Competence showed a moderate negative correlation with anger, disgust, and contempt, as well as a small negative correlation with shame. Warmth, in contrast, exhibited small negative correlations with disgust and contempt. Lastly, morality showed a moderate inverse correlation with the emotions of disgust and contempt, and slight inverse correlation with anger and shame.

Path analysis of the study variables and their effect on Social Distance: Openness to Coexistence and Tendency to Exclusion

**Figure 1.**
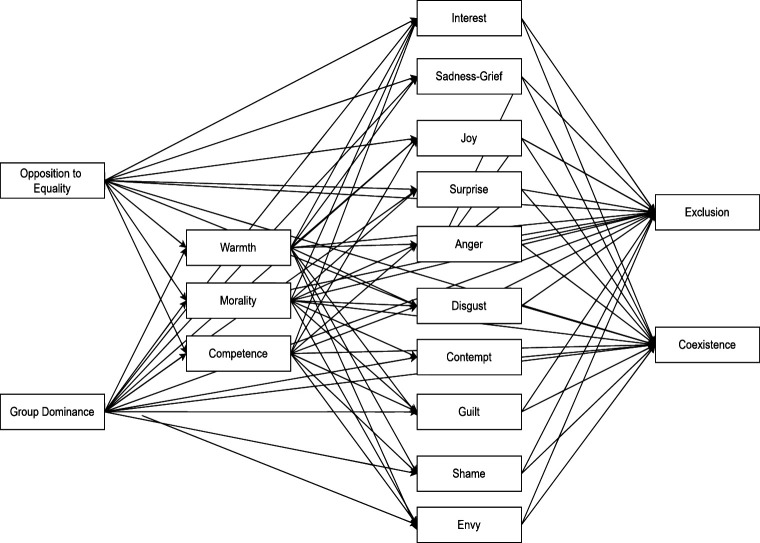
Hypothetical Model

A path analysis was conducted to comprehensively examine the relationships across the study variables. A hypothetical model was proposed to illustrate the relationships across the variables at four levels (see *Figure 1*). At the first level, the dimensions of SDO as an ideological component (opposition to equality and group dominance) are considered exogenous variables, exerting a direct influence on the other variables. stereotypes of outgroups (warmth, morality, and competence) are placed at the second level, and intergroup emotions (positive emotions, negative emotions, and sadness) at the third level. Lastly, at a fourth level, the dimensions of social distance (tendency to exclude and openness to coexistence) are considered output variables influenced by the preceding set of variables.

The goodness of fit of the proposed models was evaluated according to specific criteria outlined by Ruiz et al. (2010). Thus, the Chi-square coefficient (χ2) relative to the degrees of freedom (x2/gl) was evaluated with scores less than or equal to 3 considered indicative of good fit. In the same vein, the Bentler-Bonett comparative fit index (CFI) was evaluated with scores greater than or equal to .95 indicating a good model fit. Additionally, the Bentler-Bonett normed fit index (NFI) was considered adequate with scores greater than or equal to .9. Finally, the Steiger-Lind root mean square error of approximation (RMSEA) indicator was included with scores lesser than or equal to .08 considered indicative of an acceptable model fit.

The goodness-of-fit indicators for the hypothetical model exceed the established thresholds: χ^2^/gl = 37.73, CFI= .355, NFI= .499, and RMSEA = .3. The alternative models are detailed below. A detailed evaluation of the impact of the variables on the *social distance* dimension would be facilitated by analyzing the models and the significance of the cases. Two independent alternative models were used, each with its respective dimensions as output variables.

### Alternative Model for the Tendency to Exclusion

The goodness-of-fit indicators for the alternative model that considers exclusion tendency as the output variable were: χ^2^/gl = 3.19, CFI= .978, NFI= .97, and RMSEA = .075.

In this model, opposition to equality has an inverse effect on morality (β = –.16, p < .001). Group dominance, on the other hand, has an inverse effect on morality (β = –.23, p < .001) and a direct effect on exclusion tendency (β = .24, p < .001). From a secondary level, morality has an inverse effect on contempt (β = –.32, p < .001) and anger (β = –.33, p < .001). Finally, the dimension of exclusion tendency receives a direct effect from contempt (β = .17, p < .001) and anger (β = .11, p < .001), and an inverse effect from morality (β = –.43, p < .001).

**Figure 2.**
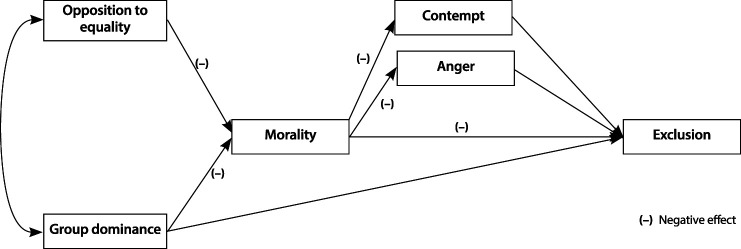
Alternative Model – Social Distance: Exclusion

### Alternative Model for Openness to Coexistence

The goodness-of-fit indicators for the alternative model for the dimension of openness to coexistence are as follows: χ^2^/gl = 1.76, CFI= .994, NFI= .987, and RMSEA = .044. In this model, opposition to equality has an inverse effect on both morality (β = –.22, p < .001) and openness to coexistence (β = –.28, p < .001). Group dominance, on the other hand, only has an inverse effect on morality (β = –.24, p < .001). At a secondary level, morality has a direct effect on joy (β = .44, p < .001) and openness to coexistence (β = .48, p < .001). Joy has a direct effect on openness to coexistence (β = .09, p < .001).

**Figure 3.**
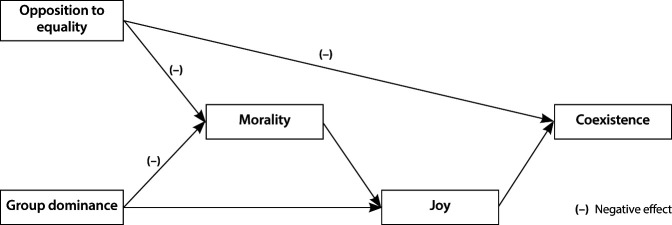
Alternative Model – Social Distance: Coexistence

## Discussion

An important observation to highlight is the sample does not exhibit a strong tendency towards social dominance in any of its dimensions. This finding is consistent with studies of similar samples of Lima adults with medium or high levels of education, who tend to show lower levels of social dominance orientation alongside more positive evaluations and orientations towards outgroups, even those perceived as being lower status (Cueto et al., 2021; Guillén et al., 2018).

Meanwhile, stereotypical perceptions of Venezuelans as warm are noteworthy, corresponding to stereotypical perceptions of Latin American groups as sociable, friendly, and helpful (Cabrera et al., 2018; Guillén et al., 2018; Gissi et al., 2019). Perceiving immigrants as competent is also important, as it relates to recognizing their ability to find employment, even informally, as national statistics indicate (Barajas et al., 2021).

Regarding intergroup emotions, the level of sadness felt for Venezuelan immigrants and the positive emotions they elicit in the sample, suggests an emotional scenario characterized by a mix of victimizing anger and pity. This condescending view has also been observed in studies with Latin American samples towards groups considered competent but still of low status (Cueto et al., 2021; Guillén et al., 2018).

In the Ecuadorian context, studies on negative emotions and attitudes toward immigration typically focus on emotions such as fear, distrust, hopelessness, anger, and anxiety, with little attention given to sadness as a negative emotion. These emotions are strongly correlated with anti-immigrant attitudes and the rejection of Venezuelan immigrants, highlighting the exclusionary sentiments that characterize the public discourse on migration more generally (Umpierrez et al., 2023; González et al., 2022).

In line with earlier research, the sample for this study indicates that younger participants are more likely to support equality and inclusion and to be less conservative (Diaz et al., 2018). Youth between the ages of 18 and 24 exhibit greater levels of empathy and respect for others, according to studies using Peruvian samples (Ipsos Peru, 2019). Specifically, regarding Venezuelan immigration, it has been found, as in the present study, that the youngest participants in the sample exhibit relatively low levels of prejudice, a better perception, and a greater openness to coexistence with Venezuelan immigrants (Alvarez & Chavez, 2018; Institute of Democracy and Human Rights of the Pontifical Catholic University of Peru [IDEHPUCP], 2020).

Regarding the associations between variables, as in previous studies (Cohrs & Asbrock, 2009; Ferreyros, 2019; Vezzali & Giovanni, 2010), and as hypothesized, higher levels of social dominance orientation are linked to a more negative view of the migrant population, as well as emotions align with aversive or hostile emotions, such as anger, disgust, and contempt. Literature suggests that these emotions are correlated with negative behaviors towards the exogroup. Specifically, emotions such as disgust and contempt are linked to increased avoidance of the exogroup (Mackie et al., 2000). This finding confirms the hypothesis proposed in this study. As aversive or hostile emotions towards the migrant population intensify, the tendency to coexist with them decreases. Conversely, the probability of acting in an exclusionary manner towards them increases.

It has been found that the emotion of guilt aroused by exogroups is connected to the authority that is ascribed to them. Thus, groups perceived as powerless are often devalued, invalidated, and excluded, thereby favoring the maintenance of social asymmetries (Sabido, 2019). This study shows that shame increases alongside social dominance, consistent with the devaluing agenda of dominant sectors toward immigrant populations of perceived lower status and power, to whom they attribute less positive stereotypical characteristics. In contrast, dominance tends to mitigate approach emotions (Cohrs & Asbrock, 2009; Ferreyros, 2019; Vezzali & Giovanni, 2010) such as joy and interest, and instead promotes avoidance and a lower perception of positive outgroup characteristics.

From another perspective, it is important to highlight that openness to coexist with the migrant population is directly associated with positive emotions and sadness, which creates an ambivalent emotional scenario. This ambivalence in emotions could be influenced by personal interactions with Venezuelan migrants, who represent a significant percentage of the sample and may generate these positive emotions, as found in previous studies (Freier et al., 2021). These positive interpersonal experiences with the Venezuelan immigrant population contrast with the negative messages about this group that are spread particularly through the media and social networks, as well as by authorities and law enforcement agencies (Menéndez, 2022; Peru 21, 2022; Sánchez, 2021).

In the case of Colombia, social perceptions of Venezuelan migrants reflect a duality: they are seen both as a threat to social and economic stability, and as victims of the crisis in Venezuela. This perception, influenced by individual experiences and stereotypes, generates a complex and varied attitude, in which Venezuelans are perceived as outsiders to the national group. This division in perception highlights the contrasting attitudes within Colombian society, where some view Venezuelans as a burden, while others recognize the need for solidarity and assistance (Perdomo et al., 2023).

According to studies pertaining to stereotypes within press media concerning Venezuelan immigration in Peru, both print and digital publications frequently link migrants to criminal activity. This approach can influence public perception, contributing to the formation of stereotypes and prejudices against this population (Regis, 2024). Thus, Freier et al. (2021) found that the Venezuelan migrant population are perceived as responsible for the increase in criminal violence, citizen insecurity, and job loss for Peruvians, as well as associated with greater informality and tax evasion, which perpetuates a negative stereotypical image of this group.

In terms of path analysis models, the χ^2^/df values of the *alternative model 1* slightly exceed the acceptable range, however, this is attributed to the fact that this estimate is highly sensitive to sample size (Hu & Bentler, 1999). Conversely, some authors accept a χ^2^/df value of less than 5 in samples larger than 300 participants (Aguilar et al., 2020).

In the identified SEM pathways, social dominance orientation plays a central role in promoting the tendency to exclude migrant populations and in hindering openness to coexistence with them. This observation correlates with both the levels of conservatism in Peruvian society, where dynamics of exclusion and differentiation prevail among ethnic and social categories (Cueto, 2017), and the precarious social conditions in which migrants are inserted, commonly associated with informality, poverty, and violence (Freier et al., 2021). According to the study, both elements would encourage people from dominant ideological sectors to associate the Venezuelan immigrant social group with unfavorable stereotyped traits and linked to fewer positive feelings regarding social integration.

The study’s findings not only highlight the negative evaluation of migrants by more conservative sectors, but also provides evidence of the presence of an ideological system and psychosocial scenarios that place the migrant population at risk of harmful effects derived from conservative attitudes, as suggested in previous studies on the topic (Cohrs & Asbrock, 2009; Ferreyros, 2019; Vezzali & Giovanni, 2010).

The differentiated effect of the SDO dimensions on the model’s output variables is a particularly interesting result exhibited by the path analysis models. It appears that opposition to equality has a subtle negative effect on the integration of immigrants, while group dominance actively promotes actions against this group. Previous studies have found that opposition to equality is associated with system justification, which implies a lack of questioning regarding the inequality suffered by marginalized sectors (Silván & Bustillos, 2007). Meanwhile, dominance is linked to endogroup favoritism, which at the intergroup level, reinforces the devaluation of the exogroup and therefore increases the tendency towards their exclusion (Jost & Thompson, 2009).

Nevertheless, the role of morality stereotypes as a central variable in understanding both the tendency to exclude and the openness to coexist emerges as one of the most notable findings of this study. This finding is partially supported by previous studies that have observed the impact of morality perception on the relative status of social groups (Espinosa et al., 2016), as well as on positive expectations about the behavior of their members, which favor interpersonal trust (Beramendi, 2014; Van Vugt & Hart, 2004). Morality is a central variable in intergroup relations, particularly in the evaluation of outgroups in Latin American countries, where this dimension often receives negative scores when evaluated as a characteristic of the national collective (Beramendi, 2014; Espinosa et al., 2016; Monsegur et al., 2014). Thus, identifying social categories that act as moral reserves within a national category appear to have a positive effect on the evaluation of subnational categories (Cueto, 2017; Espinosa et al., 2016). Therefore, the findings of this study highlight the importance of consolidating and positioning a positive perception of Venezuelan migrants, based on morality, given the potential positive effect of this stereotypical characteristic on the overall evaluation of the category.

The results obtained in this study are consistent with the findings of Romero et al. (2020) in Chile, who found that both morality and SDO can predict prejudice toward migrants. It is important to highlight that the authors emphasize a negative relationship between the specific dimensions of SDO (opposition to equality) and RWA (aggression toward authority), suggesting that individuals who identify with a social elite do not necessarily support extreme punitive measures against migrants. This finding underscores the complexity of attitudes toward migrants, indicating that, while there may be rejectionist views, they do not always translate into support for harsh policies of exclusion or punishment.

In addition, prior research has reported that the effects of emotions like disdain and anger on endogroup exclusion are similar to the effects found in the path analysis. These hostile emotions are considered agentic and promote active discriminatory behavior and exclusion (Cuddy et al., 2007; Mackie et al., 2000). Conversely, although joy, as an intergroup emotion, is traditionally associated with the evaluation of high-status groups (Espinosa et al., 2007), it is recognized as playing an important role in the active integration behavior of outgroups. Evidence in this regard suggests a positive role of positive intergroup emotions in promoting a favorable intergroup contact scenario and probably in preventing intergroup conflicts and tensions (Cueto et al., 2021).

Therefore, despite a complex scenario of social exclusion, it is important to consider that there are central elements that can facilitate greater integration and intergroup coexistence. As previously mentioned, perceived stereotypes, such as warmth and competence are highly relevant as they influence positive emotions and prosocial behavior (Fiske, 2015). Specifically, being perceived as competent could have a key role in receiving support and opportunities, which might be essential for immigrants’ adaptation (Gaucher et al., 2018). Finally, a positive perception of Venezuelans’ morality could improve their public image and generate greater social acceptance and thus mitigate xenophobia (Blouin & Zamora, 2022). However, it is important to reinforce the coordination of government and civil society actions so that integration efforts are sustained over time.

## Conclusion

The results of this study enable us to understand a historical problem from a psychosocial perspective, which has gained greater relevant in Latin America due to the recent international displacements. The study emphasizes the significant role of perceptions and evaluations of social categories as promoters or repressors of intergroup integration intentions that could trigger dynamics of inclusion or exclusion (Freier et al., 2021; Menéndez, 2022; Perú 21, 2022; Sánchez, 2021).

Additionally, the study results identify other potential factors that could promote the process of social integration. One of these factors is lowering of an orientation toward social dominance, whose positive effect could be enhanced by generational influences. It is the young people in the sample who, as in other studies, stand out for exhibiting a more critical and discerning understanding of situations of exclusion in vulnerable sectors. The authors Alvarez and Chavez (2018) and Ferreyros (2019) suggest a more inclusive approach to cultural diversity, one which contrasts with the ideologically of conservatism.

Finally, although this study focuses on understanding intergroup dynamics at the psychosocial level, it cannot be overlooked that the construction of social hierarchies and the possibilities for integrating vulnerable groups in migration contexts are also influenced by the socioeconomic conditions of migrant communities. Based on the structural and sociopolitical characteristics of host societies (Silván & Bustillos, 2007; Jost & Thompson, 2009), reflections and conclusions regarding intergroup behavior within the context of Venezuelan immigration to Lima invite us to reconsider the contribution of academia in preventing and reducing situations of social exclusion and achieving integration goals. Transformation and justice for the most vulnerable sectors of society must be pursued while acknowledging the structural and systemic limitations and challenges inherent in contexts of displacement and reception.

## Limitations

This study yields significant insight into the relationships between conservatism, intergroup dynamics, and social distance in the Venezuelan immigrant population residing in Metropolitan Lima. However, it is essential to consider several inherent limitations that may impact the generalizability of the findings. It is crucial to acknowledge that the sample utilized may not fully encapsulate the demographic composition of the Venezuelan migrant population in the region. This may potentially restrict the ability to generalize the findings beyond these specific circumstances.

Moreover, the lack of access to specific data may have constrained in-depth analysis of certain variables or aspects of the phenomenon under study. These limitations underscore the necessity for future research that addresses these issues and expands our understanding of complex intergroup dynamics in migratory contexts.
